# Early Post-operative Feeding: An Investigation of Early Functional Outcomes for Oral Cancer Patients Treated with Surgical Resection and Free Flap Reconstruction

**DOI:** 10.1007/s00455-021-10363-8

**Published:** 2021-09-24

**Authors:** Grainne Brady, Lauren Leigh-Doyle, Francesco Riva, Cyrus Kerawala, Justin Roe

**Affiliations:** 1 Therapies Department, The Royal Marsden NHS Foundation Trust, London, UK; 2 Department of Surgery & Cancer, Imperial College London, London, UK; 3 Head and Neck Unit, The Royal Marsden NHS Foundation Trust, London, UK; 4 Faculty of Health and Wellbeing, University of Winchester, Winchester, UK; 5 Department of Otolaryngology, Head and Neck Surgery, Imperial College Healthcare NHS Trust, London, UK

**Keywords:** Oral cancer, Surgery, Free flap reconstruction, Early feeding, Rehabilitation

## Abstract

Traditionally patients can remain nil by mouth (NBM) for up to 12 days after oral tumour resection with free flap reconstruction to reduce the risk of flap dehiscence, poor healing and fistulae. The literature reports that patients could on average remain an inpatient for up to 20 days post-surgery. An evaluation of the impact of a defined early oral feeding protocol was undertaken investigating functional outcomes and complications rates. We prospectively reviewed tracheostomy use, length of hospital stay, non-oral feeding status and swallowing function using the Performance Status Scale for Head and Neck Cancer (PSS-HN) within a defined early feeding protocol. Twenty-nine patients underwent surgical resection with free flap reconstruction for advanced primary oral cancer between January 2018 and December 2019. Average age was 59.5 (range 24–88). Tumour sites included oral tongue (*n* = 10), maxilla (*n* = 6), mandible (*n* = 6), floor of mouth (*n* = 5) and buccal mucosa (*n* = 2). Median time to decannulation was 7 days (range 3–20 days, *n* = 11). The majority of patients were able to tolerate at least oral fluids on day 1 post-operatively (86%, *n* = 25). In addition to oral intake, non-oral feeding was required in 90% (*n* = 26), the majority of which included a nasogastric tube (NGT) placed intraoperatively 54% (*n* = 14), others required gastrostomy 46% (*n* = 12). Median time to nasogastric tube removal was 6 days (range 3–15 days). Median length of hospital stay was 10 days (range 3–51). Mean PSS-Normalcy of Diet (NOD) score at point of hospital discharge was 36.55 (95% CI 30.9–42.2). Flap failure was noted in 3% (*n* = 1). The adoption of an early oral feeding protocol suggests that there is the potential for a shorter hospital stay and earlier swallowing rehabilitation.

## Background

In the United Kingdom, one person is diagnosed with oral cavity cancer per hour, with 8772 people treated per year [1]. The preferred treatment of oral cavity cancer remains surgery and for advanced tumours involves resection of the tumour with an appropriate safety margin and subsequent free tissue transfer reconstruction. Adjuvant radiation (with or without chemotherapy) when high-risk histopathological features are evident may also be required [2].

Like all head and neck cancer (HNC), oral cavity cancer and it’s treatment can have a significant impact on an individual’s speech/swallow. The role of the speech and language therapist (SLT) in the assessment and management of functional sequelae has been detailed in national guidelines [3]. The psychological burden on patients in the early post-operative phase has also been highlighted with the specific reference to the role of the SLT in providing support to patients to adapt to their postoperative changes and the alteration to the sense of self [4].

Historically, it has been thought that early oral post-operative feeding increases the potential for an orocutaneous fistula to develop and therefore surgeons have traditionally adopted a conservative approach and kept patients ‘nil by mouth’ (NBM) for up to 12 days following surgical resection with free flap reconstruction for oral cancer [5]. It has been conceptualised that overuse of the oral mechanism/pharynx in the early post-operative phase places excessive stress on the interface between the flap reconstruction and the mucosa leading to dehiscence and potentially the formation of a secondary fistula into the neck [5].

A recent large multicentre retrospective review of over 200 patients investigated the risk factors for free flap failure, surgical complications and the non-oral feeding period in patients treated for oral squamous cell carcinoma with reconstructive free tissue transfer reconstruction [6]. The majority of patients had floor of mouth, tongue or mandibular tumours and the majority of reconstructions included forearm fasciocutaneous flap (39.5%) or a fibular osseous free flap (30.8%). Patients previously treated for oral cancer cavity and those who have had previous head and neck radiation were excluded. Standardised swallowing outcome measures are not reported in this study.

In this study, the free flap success rate was 94.4%. A post-operative complication occurred in 101 patients (41%). Minor surgical complications (infection, bleeding, scars, partial necrosis and orocutaneous fistula which did not require surgical intervention and were managed medically) were shown to increase the free flap failure rate (OR 3.32; *p* = 0.04). A major surgical complication (orocutaneous fistula, full necrosis, infection, scars and bleeding requiring surgical intervention under general anaesthesia) was encountered in 48 patients (22.3%), and these were linked to minor surgical complications (OR 2.89; *p* = 0.004) and the use of a tracheostomy (OR 5.76, *p* = 0.002). The non-oral feeding rate at the end of the hospital stay was 28.4%, and it correlated with the presence of a tracheostomy (*p* = 0.002), as well as the major and the minor surgical complications (*p* = 0.04). Average length of hospital stay was 16.9 days. The authors concluded that post-operative care favouring early and safe oral feeding and avoiding a tracheostomy can reduce the incidence of surgical complications. The post-operative feeding protocol, including day of re-introduction/type of oral trials provided, was not described however.

A more recent large prospective cohort study included 400 patients. Two cohorts were compared including patients who remained NBM for 5 days post-surgery (‘late feeding group’) and those who commenced oral intake on day 1 post-surgery (‘early feeding group’) [5]. Two hundred patients were included in each group. No significant differences were observed in terms of age, gender, smoking and alcohol use or tumour stage between the two groups. The subtype of oral cavity tumours is not defined; however, the majority of patients had previous radiation or chemoradiation treatment and the majority of patients had composite flap reconstruction. Complications at the recipient site were noted in 8% of patients but no difference was observed in the rates of flap dehiscence (4–5%) or fistula formation (4%) between the two groups. Early feeding was associated with a statistically reduced length of hospital stay (mean 11.6 days vs 20.6 days, *p* < 0.01) [5]. It was unclear from this study the nature/type of feeding protocol used or the presence or nature of SLT input. Information regarding baseline swallowing function was also not reported and validated measures of diet texture/swallowing outcome were not used. However, it is clear that not all patients tolerated oral intake in the early post-operative days with 46% of patients commencing fluids on day 1 post-operatively and 30% concurrently starting diet. By day 3, 94% of patients were taking fluids and 84% a ‘solid’ or ‘semi-solid diet’ [5].

Other longitudinal studies have identified the prognostic factors associated with return to oral intake in the months following free tissue transfer oral cavity reconstruction. Increased risks for poor functional outcomes with resections involving the tongue, nodal disease burden, pre-operative gastrostomy dependence, postoperative fistula and dental rehabilitation have been highlighted [7]. This study highlights an early feeding protocol as a favourable prognostic factor in functional swallowing outcome [7].

Our study sought to evaluate the early functional swallowing outcomes and complications rate using a detailed and replicable early feeding protocol for patients undergoing free tissue transfer reconstructive surgery for oral cavity cancer in the primary setting.

## Methods

We reviewed the prospective outcome data collected on 29 patients who had free tissue transfer reconstruction of the oral cavity for primary oral cancer. Details recorded included the nature/site of disease, type of flap reconstruction, duration of tracheostomy, duration of hospital stay, method and duration of non-oral feeding, day of re-introduction of oral feeding, complications and Performance Status Scale for Head and Neck Cancer Normalcy of Diet (PSS-NOD) [8] subscale cores at point of discharge. The early feeding protocol including standardised baseline and outcome measures and detailed standardised dysphagia diet descriptors are reported. All patients were seen for baseline evaluation of swallowing and communication function pre-operatively. Baseline evaluation of swallowing includes as standard, assessment of oro-motor function and oral trials. Clinician rated baseline measures of swallow includes the 100 ml Water Swallow Test [8], the Performance Status Scale for Head and Neck Cancer (PSS-HN) normalcy of diet (PSS-HN NOD), eating in public (PSS-HN EIP) and understandability of speech (PSS-HN Speech) subscales [9] and the MD Anderson Dysphagia Inventory (MDADI) [10]. If there is any evidence of baseline dysphagia on clinical evaluation of swallowing, an instrumental evaluation of swallowing is performed. Given the risk of oral phase difficulties in this population, videofluoroscopy using the Dynamic Imaging Grade of Swallowing Toxicity (DIGEST) [11] is often favoured over Flexible Endoscopic Evaluation of Swallowing (FEES).

Approval for this project was obtained from the Service Evaluation Committee at the Royal Marsden NHS Foundation Trust (SE752).

## Results

Twenty-nine consecutive patients at a single tertiary cancer centre underwent reconstructive free tissue transfer for oral cancer between January 2018 and December 2019.

Patient demographics including age, sex and site and staging of disease and method of reconstruction and baseline swallowing function are summarised in Table 1. The majority of patients (68%, *n* = 19) had stage IV disease. Twenty-eight of the cases had a diagnosis of squamous cell carcinoma. One patient with high-grade sarcoma was also included. Tracheostomy was required for 11 patients with median time to decannulation at 7 days (range 3–20 days). None of our patients were gastrostomy dependent at baseline; however, 17% (*n* = 5) had a gastrostomy placed pre-operatively due to poor baseline swallowing function. Table 1 provides details of baseline level of functioning with a mean PSS-NOD score at 74.8. The PSS-NOD score ranges from 0 (nil by mouth) to 100 (no diet restrictions) indicating that many of the patients in this cohort had modified their diet at baseline and were not managing a full diet without restriction.

**Table 1:** Participant demographics, site of disease, surgical reconstruction, baseline pre-surgical PSS-NOD

	Tumour resection (*n* = 29)
Sex	M: 17 F: 12
Age (mean, range)	59.5 (24–88)
Site of disease	Oral tongue: 10
	Maxilla: 6
	Mandible: 6
	Floor of mouth: 5
	Buccal mucosa: 2
Type of flap reconstruction	Anterolateral thigh free flap: 11
	Fibula free flap: 7
	Radial forearm free flap: 6
	Medial sural artery free flap: 5
Baseline PSS-NOD	Mean: 74.8
	95%CI 63.3–86.3

All of these patients were seen for clinical bedside evaluation of swallowing on day 1 post-surgery as outlined in our early feeding protocol detailed in Table 2.

**Table 2:** Early post-operative feeding protocol

Day 1 post-operatively	1. Oromotor assessment
	2. In the event of a tracheostomy, the patient is seen for assessment of suitability for tracheostomy cuff deflation ± speaking valve placement
	3. Clinical evaluation of swallowing with sips of water ± smooth puree diet (IDDSI: 4) [12]
	4. Close working with Dietitian to inform non-oral feeding plan no NGT/NGT/gastrostomy (may be placed pre-operatively)
	*Outcome measures* PSS-NOD and understandability of speech scores, maximum interincisor opening
Days 3–5 post-operatively	1. Oromotor assessment
	2. In the event of a tracheostomy: tracheostomy weaning/decannulation
	3. Clinical evaluation of swallowing with sips of water ± smooth puree diet (IDDSI: 4) [12] ± soft and moist diet (IDDSI: 5) [12] ± soft and bite-sized diet (IDDSI: 6) [12]
	4. Close working with Dietitian to inform non-oral feeding plan? Removal of NGT
	*If concerns regarding swallowing safety—instrumental evaluation of swallowing using FEES/Videofluoroscopy with rehabilitative swallowing exercises as appropriate
	*Outcome measures* PSS-NOD and understandability of speech scores, maximum interincisor opening ± Penetration Aspiration Scale (PAS) [13]/DIGEST
Days 7–14 post-operatively	1. Oromotor assessment
	2. In the event of a tracheostomy: tracheostomy weaning/decannulation
	3. Clinical evaluation of swallowing with sips of water ± smooth puree diet (IDDSI: 4) ± soft and moist diet (IDDSI: 5) [12] ± soft and bite-sized diet (IDDSI: 6) [12] ± regular easy chew diet (IDDSI: 7 easy chew) [12]
	4. Close working with Dietitian to inform non-oral feeding plan? Removal of NGT/conversion to gastrostomy
	*If concerns regarding swallowing safety/efficiency-instrumental evaluation of swallowing using FEES/Videofluoroscopy with rehabilitative swallowing exercises as appropriate
	*Outcome measures* PSS-NOD and understandability of speech scores, maximum interincisor opening ± PAS/DIGEST

Based on clinical swallowing evaluation, the majority of patients were able to tolerate oral fluids on day 1 post-operatively (86%, *n* = 25) and continued to progress to a smooth puree (IDDSI:4)–soft and moist diet (IDDSI: 5) during their inpatient stay. A minority of patients remained on fluids only at point of discharge 7% (*n* = 2) or NBM due to persistent orocutaneous fistula 3% (*n* = 1) or total flap failure 3% (*n* = 1). Non-oral feeding was required in 90% of patients (*n* = 26), including 41% (*n* = 12) who required gastrostomy placement. Gastrostomy was placed pre-operatively in 17% (*n* = 5), and the remainder 24% (*n* = 7) were placed postoperatively. The decision for gastrostomy placement was multifactorial including multidimensional assessment of baseline evaluation of swallowing function, patient preference and the potential need for adjuvant treatment following surgery. Of note, 62% (*n* = 18) of our patients required adjuvant treatment (post-operative radiation: *n* = 16, post-operative chemoradiation: *n* = 2). In total, orocutaneous fistula was noted in 10% of patients (*n* = 3). Transient orocutaneous fistula was noted in 1 patient at 17 days post-surgery. This resolved with medical treatment (antibiotics) without needing further surgical intervention. The patient was placed NBM for a period of 7 days and then resumed soft and moist diet (IDDSI: 5). Two further patients experienced significant complications including one instance of a bleed requiring surgical intervention and one instance of failed extubation requiring tracheostomy. Both of these patients were then found to have orocutaneous fistulae on days 1 and 3 post-operatively requiring surgical intervention. In both instances, the patients had not commenced oral intake/been reviewed by the SLT prior to the discovery of the fistula. One patient (3%) experienced complete flap loss. The three patients who experienced orocutaneous fistula had T3/T4, N2b/c disease of the tongue and floor of mouth and required tracheostomy. Where a flap was lost, demarcation was noted on day 9 and was associated with a neck infection. Table 3 summarises median days for NGT removal at 7 days (range 1–19), median duration of hospital stay at 10 days (range 3–51 days) and mean PSS-NOD score at point of discharge home at 36.55 (95% CI 30.9–42.2).

**Table 3:** Number of days to NGT removal, duration of hospital stay and PSS-NOD scores at point of discharge

		*n*
Days to NGT removal	Median: 7, range 1–19 days	14
Length of hospital stay	Median: 10, range 3–51 days	29
PSS-NOD at point of discharge	36.55 (95% CI 30.9–42.2)	29

## Discussion

To our knowledge, this is the first paper reporting baseline and early post-treatment standardised swallowing outcome measures using a detailed and replicable early feeding protocol for patients undergoing free tissue transfer reconstructive surgery for oral cavity cancer in the primary setting.

In keeping with the existing literature, our cohort of 29 patients were found to have a shorter hospital stay with a median of 10 days in comparison to historical cohorts at our centre; where in the delayed feeding group, the average length of stay was 20.6 days [5]. Minor post-operative complications were noted with three patients who experienced small orocutaneous fistulae, which spontaneously resolved with medical treatment (antibiotics) and a period of NBM status. In one study reporting on risk factors for post-operative complications, the patients who experienced orocutaneous fistulae had large T3/T4 tumours with N2b/N2c neck disease and required tracheostomy [6]. One patient experienced flap loss; however, this patient had been NBM due to swallowing difficulties and had a neck infection at the time of flap loss on day 9. Post-operative infection has been described previously as a risk factor for post-operative complications including flap loss [6].

The majority of our cohort presented with advanced T3/T4 disease, and almost two thirds of our patients required post-operative radiation with a small number requiring post-operative chemoradiation. However, only 42% of our patients required a gastrostomy. Decision-making regarding this being multifaceted based on thorough multidimensional evaluation of baseline swallowing function, detailed post-operative evaluation of swallowing with the use of instrumentation, patient preference and close working with our dietetic colleagues.

This study is not without its limitations. This is a small cohort of only 29 patients with data collected at a single site with no control group for direct comparison. Patients undergoing surgical resection and free flap reconstruction in the recurrent setting were not included. As with other studies, all types of oral cancer were included [5, 6]; however, it is acknowledged that patients with tongue/floor of mouth defects may be at increased risk of orocutaneous fistula/poorer swallowing outcomes in comparison to other oral tumour subsites. A single, clinician-rated measure of swallowing function rather than multidimensional outcomes is reported for the purposes of this paper at baseline and at the point of inpatient hospital discharge.

One of the main strengths of this study is the detailed description of a replicable early feeding protocol for patients undergoing free tissue transfer reconstructive surgery for oral cavity cancer. This has been lacking in the literature to date.

## Conclusion

We describe a detailed early feeding protocol which uses repeated, validated swallowing outcome measurement, standardised dysphagia diet descriptors and the indications for clinical versus instrumental evaluation of swallowing in the early post-operative phase to guide early dysphagia rehabilitation for patients with oral cancer undergoing surgical resection with free flap reconstruction.

Given the ongoing variation in practices across the UK and beyond, further prospective data are required to further define and evaluate an evidence-based protocol for early feeding in the post-operative setting following resection of the oral cavity with free flap reconstruction. Further studies should employ robust and multidimensional functional outcomes, including patient-reported outcome measures, with pre-defined data collection timepoints to allow for more meaningful comparison between studies.
